# Effect of Peripheral Defocus Contact Lens Residual Decentration After Six Months of Wear on the Astigmatic Component in Myopic Children

**DOI:** 10.3390/diagnostics16203326

**Published:** 2026-10-13

**Authors:** Veronica Noya-Padin, Noelia Nores-Palmas, Jacobo Garcia-Queiruga, Maria J. Giraldez, Eva Yebra-Pimentel, Hugo Pena-Verdeal

**Affiliations:** 1 GI-2092 Optometry, Applied Physics Department (Optometry Area), Facultade de Óptica e Optometría, Universidade de Santiago de Compostela, 15705 Santiago de Compostela, Spain; veronicanoya.padin@usc.es (V.N.-P.); hugo.pena.verdeal@usc.es (H.P.-V.); 2 AC24 Optometry Group, Instituto de Investigación Sanitaria Santiago de Compostela (IDIS), 15706 Santiago de Compostela, Spain

**Keywords:** astigmatism, myopia management, myopia control, peripheral retinal defocus contact lens

## Abstract

**Background/Objectives**: To ascertain if the residual decentration of contact lenses with peripheral retinal defocusing geometry could be associated with alterations in the astigmatic refractive component over a six-month period of wear in children. **Methods**: A total of 39 eyes from 39 participants (mean age: 11.1 ± 1.54 years, ranging from 6 to 13 years) were included. Participants underwent an eye examination and were assigned to one of three studied lenses: multifocal contact lenses with either non-adjustable (Biofinity Multifocal) or adjustable parameters (Gentle 80 Multifocal), or a contact lens designed for myopia control (MYLO). After six months of lens wear, a refractive assessment was performed and topographic images of the eye with the contact lens fitted were taken. From these images, the decentration of each contact lens was calculated using ImageJ 1.54d software. **Results**: There was no significant difference in the decentration amount or angle between the three contact lenses studied (one-way ANOVA, both *p* ≥ 0.088). After six months of contact lens wear, there were no significant differences in astigmatic components (cylinder power and axis and J_0_ and J_45_ vectors) related to lens decentration (Wilcoxon test; all *p* ≥ 0.209). Moreover, there was no significant relationship between lens decentration (amount or angle) and changes in astigmatic components (Spearman’s correlation, all *p* ≥ 0.147). **Conclusions**: Residual contact lens decentration was observed in both adjustable and non-adjustable lenses. However, within the limitations of this study, no significant correlation was detected between lens decentration and variations in the astigmatic refractive components after six months of contact lens wear.

## 1. Introduction

Myopia has been growing in importance in recent decades due to its increasing incidence and its onset at younger ages. With an estimated one third of the global population currently affected, projections indicate that myopia will affect around half of the global population within the next 25 years [1]. Beyond the visual difficulties inherent to myopia, this refractive error is related to ocular health risks, such as an increased likelihood of retinal detachment or glaucoma, which can ultimately result in blindness. Additionally, its socioeconomic costs, driven by healthcare expenses and productivity loss, are substantial, and are expected to escalate in line with this prevalence increase [1,2]. Therefore, the development of techniques aimed at controlling myopia may hold significant potential for enhancing both individual quality of life and the overall health of the general population [2].

Among the main myopia control techniques currently in use are pharmacological approaches, such as the instillation of atropine drops, and optical approaches based on peripheral retinal defocusing [2,3]. On this second group, one of the most widely used optical control methods is contact lenses (CLs), which are based on diverse geometrical designs to effectively manage myopia. These designs include soft CLs with a peripheral power gradient, in which the power increases progressively and continuously from a central zone that provides refractive correction towards the periphery, with a more positive power. Moreover, there are designs in which the power changes more abruptly between different optical zones, such as certain bifocal or dual-focus designs. Furthermore, there are CLs that utilize specific optical profiles and/or the manipulation of higher-order aberrations to extend the depth of focus [3,4]. The efficacy of these different lens designs has been assessed in numerous investigations, evaluating both CLs specifically designed and commercialized for myopia control, as well as presbyopia CLs used “off-label” for the same purpose [5,6,7]. In both instances, regardless of the optical geometric design on which they are based, there is strong evidence of their activity in slowing the progression of myopia in children [3,5,6,7,8,9]. However, there are still unresolved questions, such as the potential impact of CL decentration on the process of emmetropization, which could lead to asymmetrical eye growth and unexpected refractive outcomes [10]. Some CLs have non-adjustable parameters, and even those with adjustable parameters may not achieve an optimal fit [11]. This may result in a decentration of the CL, potentially leading to suboptimal focusing of the rays on the retina. Further research is essential to fully understand these implications and optimize CL design or fitting procedures for better myopia management. Consequently, the present study aimed to assess if the residual decentration of different CLs with peripheral retinal defocusing geometry used for myopia control over a six-month period could be associated with a refractive alteration in the astigmatic component in children.

## 2. Materials and Methods

### 2.1. Sample and Inclusion Criteria

Before the start of this study, PS Power and Sample Size Calculations Software Version 3.1.2 (Copyright © by William D. Dupont and Walton D. Plummer, Nashville, TN, USA) was used for sample size calculation. The determination of the sample size for this study, which involves continuous response variables obtained from paired sessions, was based on the ability to detect variations in astigmatism. The prior clinical literature on astigmatism prevalence in children suggests that the mean standard deviation (SD) of repeated measures follows a normal distribution with a value of 0.56 diopters (D) [12], with a minimal clinically significant difference of 0.50 D [13]. To achieve a statistical power of 0.80 for rejecting the null hypothesis, with a Type I error probability of 0.05, a minimum sample of 12 eyes was required per group. Accounting for 25% potentially unusable images due to inadequate power ring visibility, the total sample size needed for this study was determined to be 15 on each studied group.

For inclusion in this study, participants were required to be between 6 and 13 years of age, be myopic with a refractive error between −0.25 and −5.00 D and astigmatism ≤0.75 D, and have a monocular best corrected visual acuity higher than 0.9 on a Snellen Chart at distance [14,15]. The exclusion criteria included the presence of ocular pathology or an irregular cornea, the use of ocular or systemic pharmacological treatments that could affect pupillary size or refractive status, and previous ocular surgery [14,15].

A total of 43 volunteer participants were recruited among the myopic pediatric subjects attending the Optometry Clinic of the Centre. Informed consent was obtained from the legal guardians of the minors to be included in this study. The study protocol adhered to the ethical standards of the Declaration of Helsinki and was approved by the University Bioethics Committee (approval code: USC 04/2022; approval date: 25 February 2022).

### 2.2. Study Design

The present study was designed as a longitudinal trial, involving multiple follow-up sessions as part of a myopia control protocol. In this context, the participants were fitted with CLs based on the principle of peripheral retinal defocus, evidenced as a trigger for the reduction in myopia progression in previous studies using specific myopia control CLs as well as multifocal CLs employed “off-label” [6,16,17]. The CLs employed in the present analysis with this purpose were the following: a multifocal CL with non-adjustable parameters (Biofinity Multifocal, CooperVision Inc., Pleasanton, CA, USA), a multifocal CL with adjustable parameters (Gentle 80 Multifocal, Mark’ennovy, Madrid, Spain), and a CL designed for myopia control (MYLO, Mark’ennovy, Madrid, Spain). Each CL was fitted in accordance with the manufacturer’s guidelines, with adjustments made to achieve maximum centration on the cornea when possible. The characteristics of the CLs are outlined in Table 1.

Qualified participants were scheduled to attend four sessions.

- Session 1: The clinician evaluated the keratometry and objective refraction by auto-refractokeratometer Nvision-K 5001 (Rexxam Co., Kagawa, Japan) [18], white-to-white by MYAH device (Topcon Healthcare Inc., Tokyo, Japan) [19], ocular surface health using the slit-lamp SL-D4 (Topcon Corporation, Tokyo, Japan), and the participant’s refractive status by subjective refraction with fogging technique [20]. Based on the data obtained in this session, participants were stratified by age and refractive error and randomly assigned to one of the three study CLs, after which the corresponding peripheral defocus CLs were ordered. All CLs were fitted according to the manufacturers’ recommendations.
- Session 2: The clinician checked the CL fitting by evaluating refraction over the CL, as well as their centration and movement. If the CLs were appropriated, the child was instructed on proper handling and care of the lenses, as well as the recommended time of wear (at least 8 h per day, 6 days per week) [21]. If adjustments were needed, replacement CLs were ordered, and the child was scheduled to repeat session 2.
- Session 3: After 1 week of CL wear, the clinician assessed lens management, inquired about compliance, and assessed ocular integrity.
- Session 4: Six months after session 2 (corresponding to 6 months of CL wear), the clinician reassessed the participant’s refractive status and captured an image of the CL in the eye with the MYAH device.

**Figure 1:**
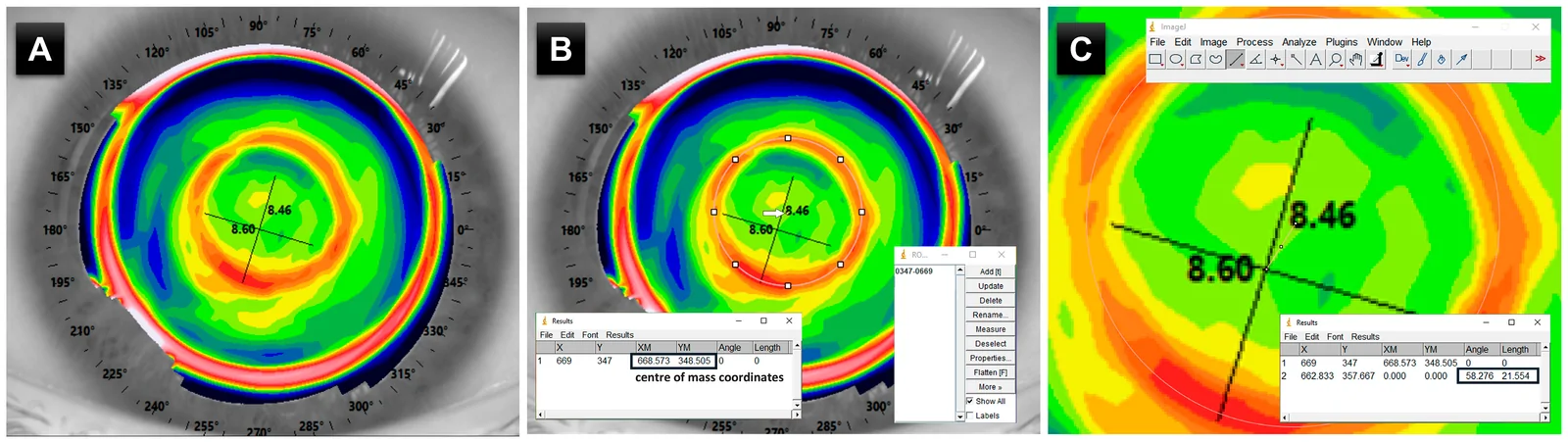
Process of measuring the amount and angle of contact lens decentration. (**A**) The image, captured using a topographer, depicts the center of the cornea. (**B**) The measuring circle was plotted on the best-defined defocus ring of the map. With the coordinates of the center of mass of the aforementioned circle, the central point (indicated by the arrow) was marked. (**C**) A line was drawn from the marker indicated by the topographer to the calculated marker at point (**B**). The coordinates of the center of mass of the circle and the values of the angle and amount (length) of contact lens decentration are indicated in lines 1 and 2, respectively, of the result box.

After session 4, two additional clinicians evaluated each image to ascertain the amount and angle of residual decentration of the CL using ImageJ 1.54d open-source software (National Institutes of Health, Bethesda, MD, USA) [22]. To this end, the image of the topographic map was extracted from the topographer, with the inclusion of a mark in the corneal center as a default feature (Figure 1) [11].

**Figure 2:**
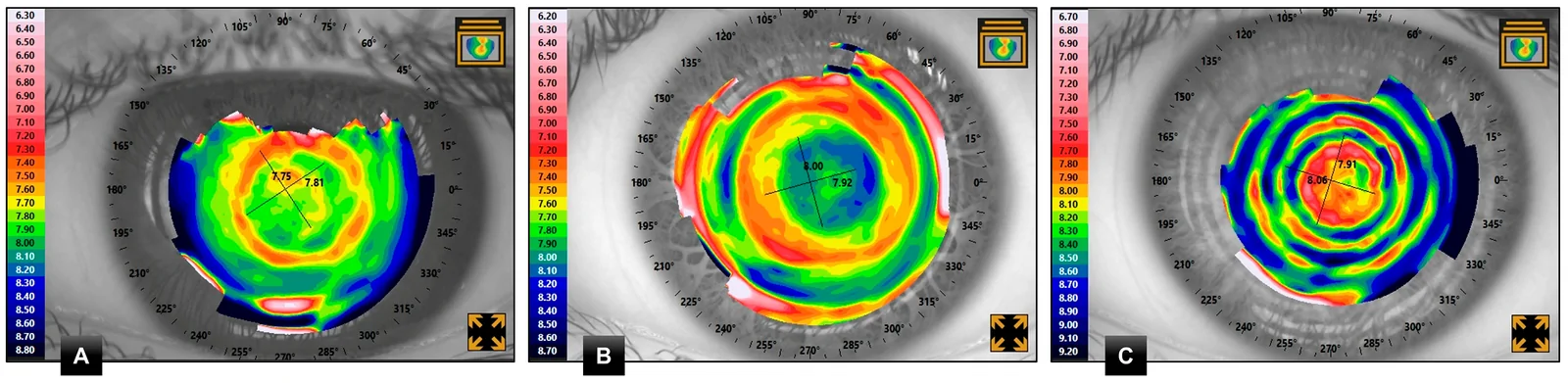
Examples of topographical images of the contact lenses fitted in the eye, showing the areas of defocus. The lenses are identified as follows: (**A**) Biofinity Multifocal contact lens, (**B**) Gentle 80 Multifocal contact lens, and (**C**) MYLO contact lens.

As the defocus zones of the CL exhibit a variation in optical power, these are discernible on the topographical map (Figure 2). These regions are represented by concentric rings extending from the central lens position, which were employed in assessing CL displacement. Firstly, a circle was drawn around the best-defined defocus ring on the map, and the coordinates of the center of mass of the circle were calculated in order to determine the center point of the CL (Figure 1B). Subsequently, a marker was placed at these coordinates (Figure 1B, arrow). A line was then drawn from the center of the cornea (indicated in the image by the topographer) to the marker representing the center of the CL (Figure 1C). The amount and angle of CL residual decentration were obtained from the length and angle characteristics of the line (Figure 1C). To ensure the precision of the measurements and to prevent any potential bias due to imaging issues, only those topographies that permitted the accurate mapping of the CL center were incorporated into the final analysis.

### 2.3. Statistical Analysis

For statistical purposes, the refractive values measured in sessions 1 and 4 were transformed into vectorial components at 0° (J_0_) and 45° (J_45_) using the following equations [23]:

J_0_ vector = −(Cylinder power/2) × cos (2 × Axis of cylinder), (1)

J_45_ vector = −(Cylinder power/2) × sin (2 × Axis of cylinder), (2)

Data analysis was performed with SPSS statistical software v.29.0 for Windows (SPSS Inc., Chicago, IL, USA). The significance level for all analyses was *p* ≤ 0.050. Prior to analysis, the normal distribution of the data was checked using the Shapiro–Wilk test [24].

To assess the reliability of the lens decentration measurements, both repeatability and reproducibility were evaluated. Repeatability was assessed by comparing two independent measurements performed by the same observer, while reproducibility was assessed by comparing measurements obtained independently by two observers. Quantification of these parameters was undertaken using the intraclass correlation coefficient (ICC), calculated using a two-way mixed-effects model with absolute agreement for single measurements, with 95% confidence intervals (95% CI). ICC values of less than 0.75 are indicative of poor reliability, values between 0.75 and 0.90 demonstrate good reliability, and values above 0.90 indicate excellent reliability. The standard error of measurement (SEM) and the minimum detectable change at the 95% confidence level (MDC95) were calculated in order to quantify the magnitude of measurement error and the smallest difference needed to confirm actual changes, respectively. The pooled standard deviation (SDpooled), required for the calculation of these parameters, was first determined. Equations (3)–(5) were applied to derive these variables [25,26].

SD pooled = √ [(SD_1_^2^ + SD_2_^2^)/2], (3)

SEM = SD pooled × √ (1 − ICC),(4)

MDC95 = SEM × 1.96 × √ 2,(5)

Paired *t*-tests were used as complementary analyses to examine systematic mean differences between repeated measurements. These evaluations were interpreted as assessments of mean-level bias rather than as measures of agreement.

The amount and angle of CL decentration showed a normal distribution (Shapiro–Wilk, *p* ≥ 0.054), while the other variables showed a non-normal distribution (Shapiro–Wilk, all *p* ≤ 0.001). The one-way analysis of variance (ANOVA) was employed to determine if there was a significant difference in the amount of displacement between the various CLs. A Wilcoxon signed rank test was used to assess differences in cylinder power, cylinder axis, and J_0_ and J_45_ vectors between sessions 1 and 4. The relationship between the amount and angle of decentration and the differences in refractive values between sessions 1 and 4 were assessed using Spearman’s correlation test. Correlation among variables was described as weak (0.20–0.40), moderate (0.41–0.60), good (0.61–0.80), or strong (0.81–1.00) [27]. To assess whether these relationships differed according to lens design, general linear models were fitted for changes in J_0_ and J_45_, including decentration amount, lens type, and their interaction, to assess whether lens type modified these relationships.

The units of measurement for decentration were obtained in pixels and converted to millimeters, with 1 pixel corresponding to 0.025 mm.

## 3. Results

Based on the inclusion criteria, the final total sample consisted of 39 right eyes from 39 volunteers (18 males and 21 females) with a mean age ± SD of 11.1 ± 1.54 years (ranging from 6 to 13 years). Of these, 12 participants wore the multifocal CLs with non-adjustable parameters, 12 wore the multifocal CLs with adjustable parameters, and 15 wore the CLs specifically designed for myopia control. Of the initial cohort of 43 participants, four were excluded as the CL center was not visible in the topography; hence, the measurement of their residual decentration in ImageJ was not possible. The average time between the first day of CL wear (session 2) and the last appointment (session 4) was 182.5 ± 3.85 days, ranging from 175 to 190 days. The refractive characteristics of the final sample at baseline session (session 1) showed a mean spherical power of −2.17 D (ranging from −0.25 to −4.50 D), and in those who also had astigmatism, a mean cylindrical power of −0.45 D (ranging from −0.25 to −0.75 D) and a mean cylindrical axis of 113° (ranging from 10° to 180°). The baseline characteristics, disaggregated by lens type, are displayed in Table 2. No significant differences were observed in the baseline characteristics of the three lens groups (one-way ANOVA, all *p* ≥ 0.315). At the final session (session 4), the mean spherical power was −2.28 D (ranging from −0.25 to −4.75 D), and in those who also had astigmatism, the mean cylindrical power was −0.50 D (ranging from −0.25 to −1.00 D) and the mean cylindrical axis was 111° (ranging from 5° to 180°).

A test for homogeneity of the amount and angle of decentration among the three CL types was performed to determine whether the data could be analyzed collectively. The analysis showed no significant differences (one-way ANOVA, both *p* ≥ 0.088), allowing data pooling for subsequent analyses. The decentration angle of the CLs was predominantly temporal/inferior (51.28%), followed by entirely temporal (28.21%), temporal/superior (15.39%), and one case each of inferior/nasal (2.56%) and entirely inferior (2.56%). The amount and angle of decentration of the CLs are displayed in Table 3.

### 3.1. Repeatability and Reproducibility

Repeatability differed according to the measurement component. Regarding the amount of CL decentration, the mean values in the first and second assessments were 28.86 ± 12.85 pixels and 29.53 ± 12.96 pixels, respectively, equivalent to 0.715 ± 0.318 mm and 0.732 ± 0.321 mm. The ICC was 0.923 (95% CI, 0.858–0.959), indicating excellent repeatability. The SEM was 3.58 pixels (0.089 mm), and the MDC95 was 9.93 pixels (0.246 mm). For angle measurements, the mean values obtained in the first and second assessments were 197.25 ± 31.29° and 192.36 ± 47.51°, respectively. The ICC was 0.472 (95% CI, 0.187–0.683), indicating poor repeatability. The SEM was 29.23°, and the MDC95 was 81.03°.

Reproducibility was assessed using both measurements obtained by observer 1 as separate comparisons with observer 2. For the amount of lens decentration, the mean value for observer 2 was 29.98 ± 11.56 pixels (0.743 ± 0.287 mm). Compared to the first measurement of observer 1, an ICC of 0.899 (95% CI, 0.816–0.945) was obtained, along with a SEM of 3.89 pixels (0.096 mm) and an MDC95 of 10.77 pixels (0.267 mm). Compared to the second measurement of observer 1, the ICC was 0.927 (95% CI, 0.865–0.961), with a SEM of 3.32 pixels (0.082 mm) and MDC95 of 9.20 pixels (0.228 mm). Consequently, the measurement of the amount of CL decentration exhibits good-to-excellent reproducibility. As observed in the repeatability analyses, greater variability was found for the angular component. The mean value of angular decentration for observer 2 was 195.01 ± 37.93°. The comparison between the first measurement from observer 1 and observer 2 yielded an ICC of 0.216 (95% CI, −0.110 to 0.497), a SEM of 30.78°, and an MDC95 of 85.33°, indicating poor reproducibility. However, when the second measurement from observer 1 was compared with observer 2, the ICC was 0.842 (95% CI, 0.720–0.914), with a SEM of 17.09° and an MDC95 of 47.36°, indicating good reproducibility and lower absolute measurement error.

The amount of lens decentration demonstrated consistently high intra- and inter-observer reliability, whereas reliability estimates for the angular component were less consistent. Paired *t*-tests revealed no evidence of systematic mean differences between repeated or inter-observer measurements for either the amount or angle of CL decentration (paired *t*-test, all *p* ≥ 0.209).

### 3.2. Alterations in the Astigmatic Refractive Component

The analysis conducted to assess potential changes in the astigmatic component after six months of CL wear showed no significant differences, either when evaluating cylinder power and axis directly (Wilcoxon test, both *p* ≥ 0.233) or when analyzing their vectorial components, J_0_ and J_45_ (Wilcoxon test, both *p* ≥ 0.209). The cylinder axis was retained for clinical description, whereas inferential analyses of astigmatic change were primarily based on the J_0_ and J_45_ vector components, which account for both cylinder power and axis. Despite the absence of statistically significant variations in any astigmatic parameters associated with residual CL decentration when comparing sessions 1 and 4, an evaluation was conducted to ascertain any potential correlation between changes in astigmatic components and CL decentration. For this purpose, the difference between session 1 and 4 was calculated for the variables of the cylinder, the axis and their vectors, thereby generating new variables. These variables were then evaluated in conjunction with the amount and angle of decentration of the CLs using Spearman’s correlation test. Spearman’s correlation tests revealed no significant relationships between the amount of CL decentration and changes in J_0_ or J_45_ vectors (Spearman’s correlation, both *p* ≥ 0.442). Exploratory analyses using cylinder power and axis values similarly showed no significant correlations with the amount of CL decentration (Spearman’s correlation, both *p* ≥ 0.147). In addition, exploratory analyses involving the decentration angle yielded no statistically significant correlations with changes in cylinder power, axis and J_0_ and J_45_ vectors (Spearman’s correlation, all *p* ≥ 0.353). Given that conventional Spearman correlation does not account for the circular nature of the decentration angle, these analyses were considered exploratory and were not used as the primary basis for interpretation. General linear models, including decentration amount, lens type, and their interaction, provided no evidence that lens type modified these relationships (general linear model, all *p* ≥ 0.125). The pooled analyses were therefore retained as the primary overall estimates.

Consequently, there is neither a significant overall change in astigmatism nor a tendency for it to change in relation to the magnitude of decentration of peripheral retinal defocus CLs administered to pediatric subjects. Exploratory analyses based on the decentration angle also yielded no significant correlations.

## 4. Discussion

The correct centration of CLs is fundamental to ensuring both the wearer’s comfort and optimal visual efficiency. Inadequate centration can result in undesirable outcomes, including reduced visual acuity or ocular discomfort [28]. This is particularly relevant when the CL has additional functions beyond being purely refractive, such as corneal shaping in orthokeratology or the reduction in myopic progression, such as in CLs designed to control myopia [28]. Although the participants in this study were fitted with peripheral retinal defocus CLs for the purpose of myopia control, the objective of this research was not to evaluate the efficacy of myopia control itself. Instead, this study aimed to ascertain if the residual decentration of CL with peripheral retinal defocusing geometry could be associated with alterations in the astigmatic refractive component over a six-month period of wear in children. Three different CLs were employed: a multifocal CL with non-adjustable parameters (Biofinity Multifocal), a multifocal CL with adjustable parameters (Gentle 80 Multifocal), and a CL designed for myopia control with adjustable parameters (MYLO). While all lenses demonstrated an adequate clinical fit, directly comparable effective optical-zone dimensions were not available for the three designs. Consequently, residual decentration could not be normalized to optical-zone diameter, and its exact effect on the power distribution across the pupil could not be determined.

Although the reliability assessment of the measurement procedure was not the primary objective of this study, the evaluation of the repeatability and reproducibility of the lens decentration measurements was relevant to determining the robustness of the procedures used to quantify residual lens positioning. The amount of CL decentration exhibited good-to-excellent repeatability and reproducibility, thereby supporting the consistency of the procedure. The angular component exhibited greater variability. Consequently, while the angular analysis provided complementary information on the direction of lens displacement, small-to-moderate differences in angular orientation may reflect measurement variability and should be interpreted with greater caution.

Contrary to what was expected, no significant disparity in residual decentration amount was observed between CLs with non-adjustable and those with adjustable parameters. This may be attributed to the mechanical properties of the CL, which are influenced by the composition of the lens materials [29]. The most notable differences between the materials of the three CLs under consideration are the water content and the modulus of elasticity. The Filcon 2 and Filcon 5B CLs exhibit high water content and a low modulus of elasticity, whereas the Comfilcon A lens displays medium water content and a medium modulus of elasticity [30]. Both parameters are related and play a significant role in CL fitting [31,32]. Furthermore, it was noted that the majority of CLs exhibited temporal and/or inferior decentration, a finding that is consistent with that observed by Rizzo et al. [11] for the MYLO CL. Despite the differences in terms of the material properties, fitting parameters and optical design of the CLs studied, the interaction analyses provided no evidence that lens type modified the relationship between residual decentration amount and changes in J_0_ or J_45_. This finding supports the presentation of the pooled analysis as an overall estimate.

In examining the impact of CL decentration on the astigmatic component, the present study revealed no significant differences. In contrast, Li et al. [10] observed asymmetric elongation at the periphery of the eyeball based on the decentration of orthokeratology CLs. The assessment of refraction in the aforementioned study was conducted as a spherical equivalent; therefore, it remains unclear if this asymmetry is related to modification of the astigmatic component. Previous studies have investigated whether the decentration of an orthokeratology CL could influence the efficacy of myopia control. It has been proposed that myopia control may be modified by the effect of decentration on the peripheral refraction of the eye and by the increase in higher-order aberrations [33]. However, this is a contentious issue, with some findings indicating more effective myopia control when using decentered orthokeratology CLs [34,35] and other findings indicating no difference [36].

In relation to peripheral refraction, it was studied if the decentration associated with eye or head movement could generate refractive changes during the wearing of hydrophilic CLs. Lens decentration was observed during eye rotation, yet this movement was insufficient to yield discernible alterations in the peripheral astigmatic component (J_0_ and J_45_ vectors) [37]. The lack of differences in peripheral refraction lends support to the findings of the present study.

Regarding this study’s limitations, one such limitation is the relatively short follow-up period of six months, which may not reveal long-term refractive changes. Myopia control studies predominantly employ a longer follow-up period, typically exceeding one year [6,7,8]. Therefore, studies with a longer follow-up period are needed to obtain stronger evidence to confirm the findings. Additionally, the sample size, while adequate for the scope of this study and the sample size calculations, could be expanded in future research to increase the generalizability of the findings. The sample size was calculated to detect a within-subject change of 0.50 D in astigmatism, based on the threshold predefined from the literature. The mean cylinder change observed over 6 months was −0.05 D. Therefore, although this study was designed to detect changes of a predefined, clinically relevant magnitude, it was not powered to detect changes as small as the observed mean difference. Moreover, this study did not include a control group wearing single-vision CLs. Therefore, it cannot be determined whether a similar evolution of the astigmatic refractive components would have occurred in children wearing conventional lenses. Accordingly, the present findings indicate that no significant correlation between residual lens decentration and changes in the astigmatic refractive components was detected in this sample during the six-month study period. Related to the assessment of CL decentration, this was determined from two-dimensional images using manually identified anatomical and lens reference points in ImageJ. Consequently, the measurements may have been influenced by factors such as image quality, acquisition conditions and the observer-dependent placement of the measurement points. Furthermore, the movement of the CL in response to changes in gaze and blink phases may also influence the recorded position of the lens. Future studies should also consider using other CL designs or materials to clarify whether residual decentration during fitting influences peripheral refraction or astigmatism, as well as its potential role in myopia control.

Given the prevalence of myopia and the associated concerns about its progression, further research is required to evaluate the impact of current methods for controlling it. In the case of CLs, it is pertinent to ascertain the effects of potential residual decentration.

## 5. Conclusions

The results of previous studies mentioning asymmetric growth of the eyeball may suggest potential concerns regarding changes in the astigmatic component. In the context of the present study, no variation in the astigmatic component was observed, nor was there any tendency for it to change. It may therefore be concluded that within the limitations of the present study, no significant correlation was detected between the residual decentration of myopia control hydrophilic CLs and variations in the astigmatic refraction after six months of CL wear.

## Figures and Tables

**Table 1:** Parameters of the CLs employed during this study. CD: Center distance.

	Biofinity Multifocal	Gentle 80 Multifocal	MYLO
Manufacturer	CooperVision	Mark’Ennovy	Mark’Ennovy
Material Name	Comfilcon A	Filcon 2	Filcon 5B
Material	Silicone-hydrogel	Hydrogel Ori:gen tech	Silicone-hydrogel
Replacement	Monthly	Monthly	Monthly
Oxygen Permeability (Dk)	128	60	60
Water content	48%	80%	75%
Modulus (MPa)	0.75	0.13	0.33
Design	Peripheral Add Design	Peripheral Add Design	Extended Depth of Focus
Add power	+2.00 CD	+2.00 CD	+1.50
Base curves (mm)	8.60	7.10 to 9.80 (0.30 increments)	7.10 to 9.80 (0.30 increments)
Diameters (mm)	14.00	13.00 to 16.00 (0.50 increments)	13.50 to 15.50 (0.50 increments)

**Table 2:** Baseline characteristics disaggregated by lens. D: Diopters.

Characteristics	Biofinity Multifocal	Gentle 80 Multifocal	MYLO	*p*-Value
*n*	12	12	15	-
Age (years)	11.59 ± 1.46	11.04 ± 1.81	10.88 ± 1.39	0.483
Sphere (D)	−1.75 ± 0.75	−2.37 ± 1.43	−2.33 ± 1.41	0.392
Cilinder (D)	−0.17 ± 0.25	−0.31 ± 0.26	−0.18 ± 0.26	0.315
Axis (°)	51.25 ± 67.96	65.00 ± 68.59	50.33 ± 71.00	0.839
J_0_ vector	0.01 ± 0.09	−0.03 ± 0.13	−0.03 ± 0.12	0.603
J_45_ vector	−0.03 ± 0.11	0.04 ± 0.16	−0.01 ± 0.10	0.364

**Table 3:** Amount and angle of decentration disaggregated by lens.

CL Decentration	Amount (mm)	Angle (°)
Biofinity Multifocal	0.76 ± 0.24	202.44 ± 34.99
Gentle 80 Multifocal	0.55 ± 0.32	202.30 ± 37.83
MYLO	0.81 ± 0.34	189.06 ± 21.32
Pooled CL	0.72 ± 0.32	197.25 ± 31.29
*p*-value	0.088	0.445

## Data Availability

The data presented in this study are available on request from the corresponding author due to privacy restriction.

## Abbreviations

The following abbreviations are used in this manuscript:

ANOVA	One-way Analysis of Variance
CD	Center Distance
CL	Contact Lens
D	Diopters
